# Supplementary material for: Pleistocene terrestrial warming trend in East Asia linked to Antarctic ice sheets growth
Source: Nat Commun. 2025 Sep 10;16:8258. doi: 10.1038/s41467-025-63331-3 (PMC12423306; doi:10.1038/s41467-025-63331-3)
Supplement: Supplementary file 1 — Supplementary Information [file 41467_2025_63331_MOESM1_ESM.pdf]

Supplementary Information for

"Pleistocene terrestrial warming trend in East Asia linked to Antarctic Ice Sheets growth"

Supplementary Discussion

Long Pleistocene terrestrial temperature records beyond East Asia

In tropical Africa, a 1.3-Myr-long temperature record inferred from TEX_{86} at Lake Malawi[1] shows an approximate 2.9 ± 1.5 °C/Myr warming trend ($p < 0.01$; 95% confidence interval = 0.2–5.9) during 1.3–0.6 Ma. However, for this record, it might be argued that the TEX_{86} paleothermometer might be complicated by the ill-defined ecology of freshwater *Thaumarchaeota*, although some samples affected by non-Thaumarchaeota contribution and soil input have been rejected for analysis[1]. At higher latitudes, long and continuous temperature records are especially scarce. The biogenic silica (BioSi) record from Lake Baikal[2], which probably tracks temperature fluctuations in high-latitude continental Eurasia, shows no obvious trend from 1.8 Ma to 0.6 Ma. However, it is generally believed that the response of BioSi to temperature is non-linear and qualitative, especially due to the significant dissolution effect during glacial period[3]. Therefore, only the interglacial trend might be reliable. Further north at Lake El'gygytgyn, Far East Russia, while regional aridification increased during the MPT, no significant warming or cooling trend was observed based on brGDGTs[4]. High-resolution, pollen-based Pliocene-Pleistocene temperature record has also been reported for this lake[5], but data from the critical 1.8–0.6 Ma period is discontinuous and scarce.

Supplementary Methods

CO_2 stack

We compiled a new CO_2 stack by normalizing Pleistocene long CO_2 reconstructions to a reference record[6]. The selection criteria for long records include: (i) the records should be vetted and modernized by the CenCO2PIP Consortium[7], (ii) the records should be longer than 1 Myr, (iii) the data are relatively evenly distributed with an average resolution < 0.5 Myr, and (iv) the time interval should overlap any portion of the 1.2–0.6 Ma period.

Totaling 11 records meet the requirements, and they can be briefly divided into 3 types according to the reconstruction methods and archives: (i) 3 records inferred from carbon isotopes of terrestrial materials ($\delta^{13}C_{terrestrial}$), including leaf wax $\delta^{13}C$ preserved at IODP U1446[6], soil carbonate $\delta^{13}C$ in eolian deposits on the Chinese Loess Plateau[8], and the $\delta^{13}C$ of global terrestrial C3 plant remains[9] (Supplementary Fig. 13b), (ii) 3 records reconstructed by boron isotopes in planktic foraminifera *G. ruber*, *G. sacculifer*, and *T. trilobus* at ODP Sites 999A, 668, and 926[10-12] (Supplementary Fig. 13c), and (iii) 5 records calculated from the carbon isotopes of marine phytoplankton biomarkers ($\delta^{13}C_{phytoplankton}$) at ODP Sites 806, 925, 999A, and 1241[11,13-15] and DSDP467[16] (Supplementary Fig. 13d). Note that for some records estimates were recalculated where needed and possible, and age models were revised where new evidence was readily accessible by the CenCO2PIP Consortium[7].

We set Yamamoto et al.'s 1460-kyr high-resolution CO_2 record[6] as the reference record, due to its high consistency to the 805-kyr CO_2 history documented in Antarctic ice cores[17] but the former has a longer time span. To be more precise, this record was first normalized to the ice core data, although the offset after this adjustment is neglectable (0.4±3.1 ppm). Subsequently, other CO_2 records were interpolated to 1-kyr resolution and then normalized to the adjusted record of Yamamoto et al.[6] based on the overlapping period. Finally, the 11 normalized records were averaged to generate a CO_2 stack (Supplementary Fig. 13e). We believe that although each record might have inherent weaknesses, the long-term trend of our stack which would mitigate these uncertainties shall be robust. The new CO_2 stack based on vetted CO_2 records suggest that atmospheric CO_2 concentration has slightly decreased during 2.6–1.8 Ma, and this trend then stalled during the latter Pleistocene interval.

Numerical simulations

We utilized the coupled atmosphere-ocean-sea ice-land surface climate model Community Earth System Model (CESM1.2) developed by the National Center for Atmospheric Research to supplement inferences that we made based on geological records. The model consists of the community atmosphere model version 4 (CAM4), the Community Land Model version (CLM4), the Community Ice CodE (CICE) and the Parallel Ocean Program version 2 (POP2). Its horizontal resolution is 0.9° latitude x 1.25° longitude and has 26 layers in the vertical direction. The ocean and sea ice models share the same horizontal grid with an approximate resolution of 1°, and the ocean model has 60 layers in the vertical. The CESM has already been widely used to investigate climate impacts of ice sheets[18,19].

To evaluate the responses of global climate to the expansion of AIS, two experiments have been performed respectively, including the preindustrial (PI) control experiment and the AIS area sensitivity experiment. The boundary conditions for PI experiment are in accordance with the protocols given by PMIP3[20], and the AIS configuration in the PI experiment is used to represent a relatively large area of AIS. The AIS experiment is modified based on the PI experiment. In the AIS experiment, only artificially shifting AIS area southwards by 5 degrees to represent a relatively small area of AIS. Therefore, the influence of AIS area expansion on climate can be investigated by comparing PI control experiment with AIS experiment. The two experiments are both integrated for 500 model years, and the monthly mean outputs of the last 15 years in simulation were used.

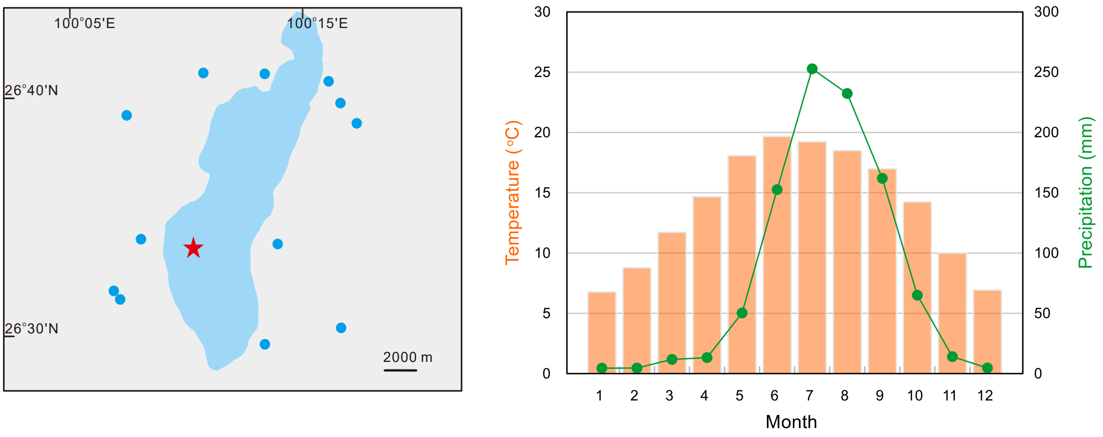

Supplementary Figure 1. **Sampling sites for soils surrounding the Heqing paleolake and monthly mean air temperature and precipitation variations.** The blue area indicates the Heqing paleolake, and the red star and blue dots indicate sites for core HQ and surface soils, respectively. Monthly mean air temperature and precipitation data were monitored by the Heqing meteorological station.

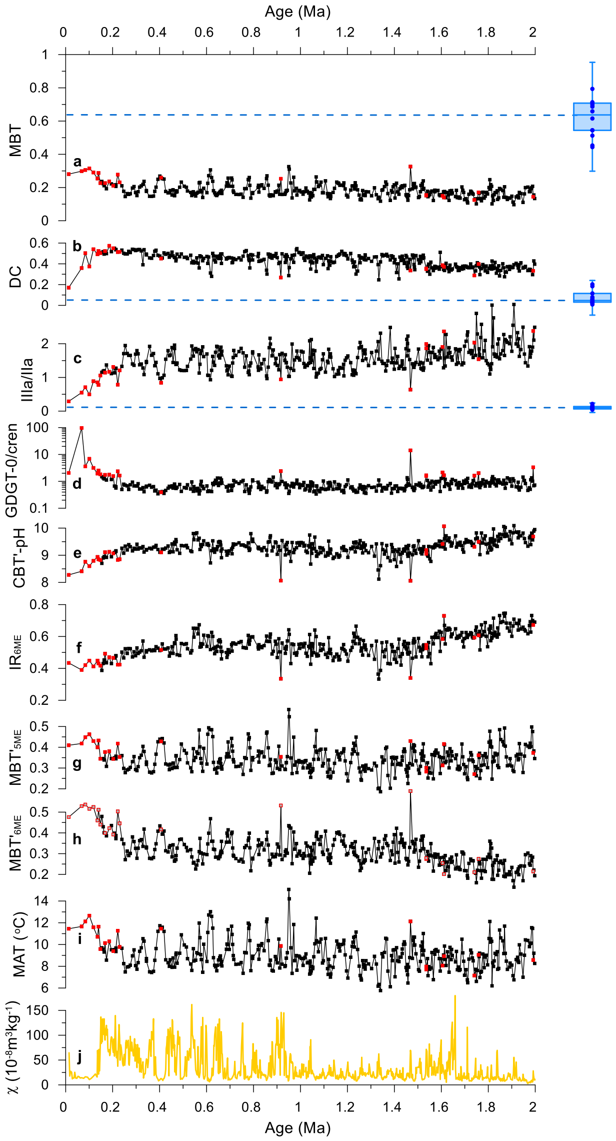

Supplementary Figure 2. **Downcore profiles of selected GDGT-derived proxies and magnetic susceptibility in the 2-Myr sediment sequence of the Heqing paleolake. a** methylation index of branched tetraethers (MBT). **b** Degree of cyclization (DC). **c** IIIa/IIa ratio. **d** GDGT-0/crenarchaeol (GDGT-0/cren) ratio. **e** Reconstructed pH based on CBT', using the calibration of Russell et al.[21]. **f** Isomer ratio (IR_{6ME}). **g** MBT'$_{5ME}$. **h** MBT'$_{6ME}$. **i** Reconstructed MAT. **j** Magnetic susceptibility (χ) record[22]. Red points indicate GDGT samples with IIIa/IIa < 0.92 or GDGT-0/cren > 1.5, which were excluded for quantitative temperature reconstruction at Heqing. Box-and-whisker plots in **a-c** indicate proxy distributions for surrounding soils (center lines, median; box limits, upper and lower quartiles; whiskers, 1.5x interquartile range; blue dots, data points).

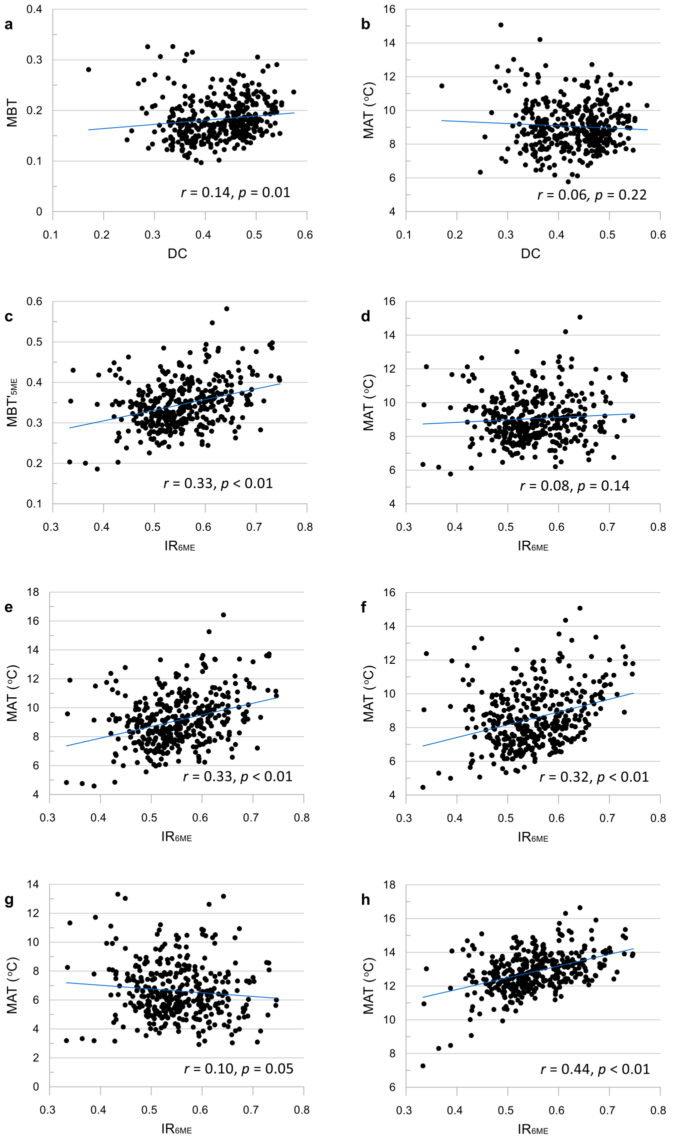

Supplementary Figure 3. **Relationships between selected GDGT proxies in the HQ core. a** Degree of cyclization (DC) versus methylation index of branched tetraethers (MBT). **b** DC versus reconstructed mean annual temperature (MAT) based on the calibration which can mitigate the mixing effect of 5-methyl and 6-methyl isomer sets[23]. **c** The ratio of 6- over 5-methyl brGDGTs (IR_{6ME}) versus MBT'_{5ME}. **d** IR_{6ME} versus reconstructed MAT based on the calibration which can mitigate the mixing effect of 5-methyl and 6-methyl isomer sets[23]. **e** IR_{6ME} versus reconstructed MAT based on the global Bayesian temperature calibration for lacustrine brGDGTs[24]. **f** IR_{6ME} versus reconstructed MAT based on the full set global lacustrine calibration by Raberg et al.[25]. **g** IR_{6ME} versus reconstructed MAT based on the tropical multivariate linear regression calibration[26]. **h** IR_{6ME} versus reconstructed MAT based on the Index 1 calibration for lakes from East Africa and Southwest China[27].

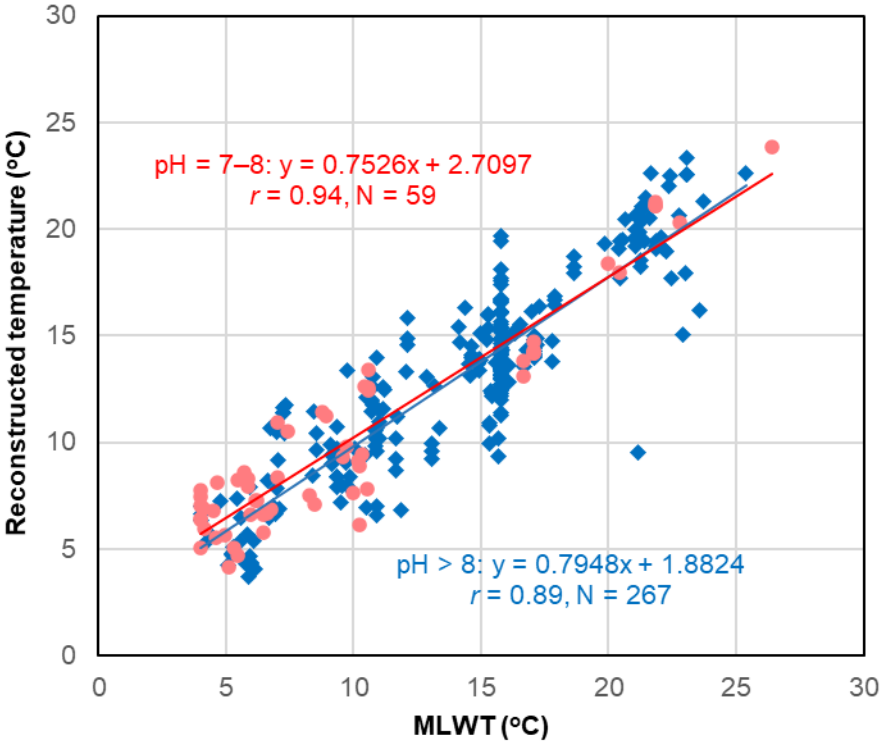

Supplementary Figure 4. **Regressions of brGDGT-inferred temperature against mean lake water temperature (MLWT) in the sediments of global freshwater lakes**[21,24,25,27-38]**.** Pink dots: lake water pH = 7–8; Blue diamonds: lake water pH > 8.

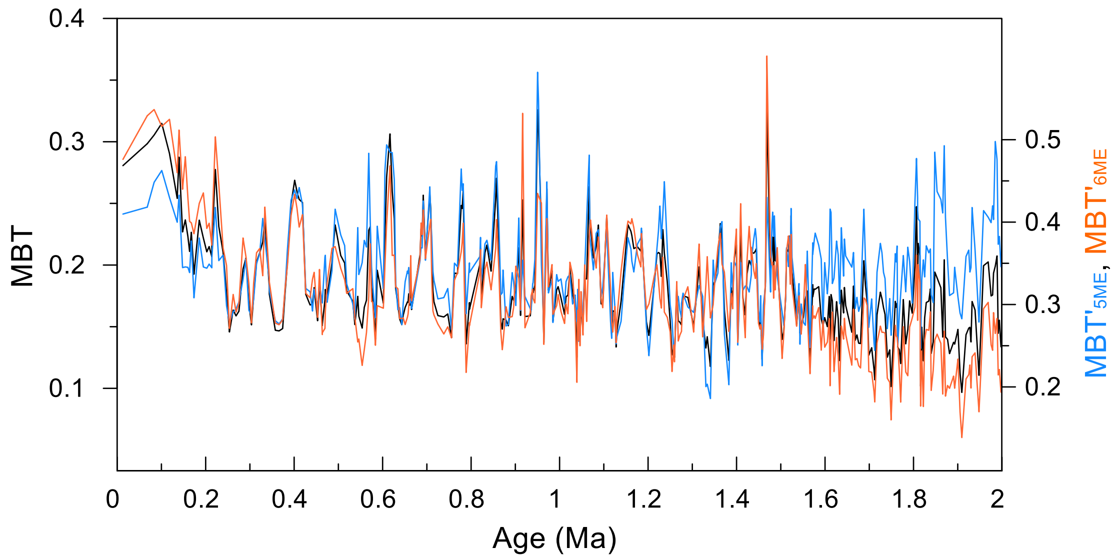

Supplementary Figure 5. **Variations of MBT, MBT'$_{5ME}$ and MBT'$_{6ME}$ in the 2-Myr sediment sequence of the Heqing paleolake.**

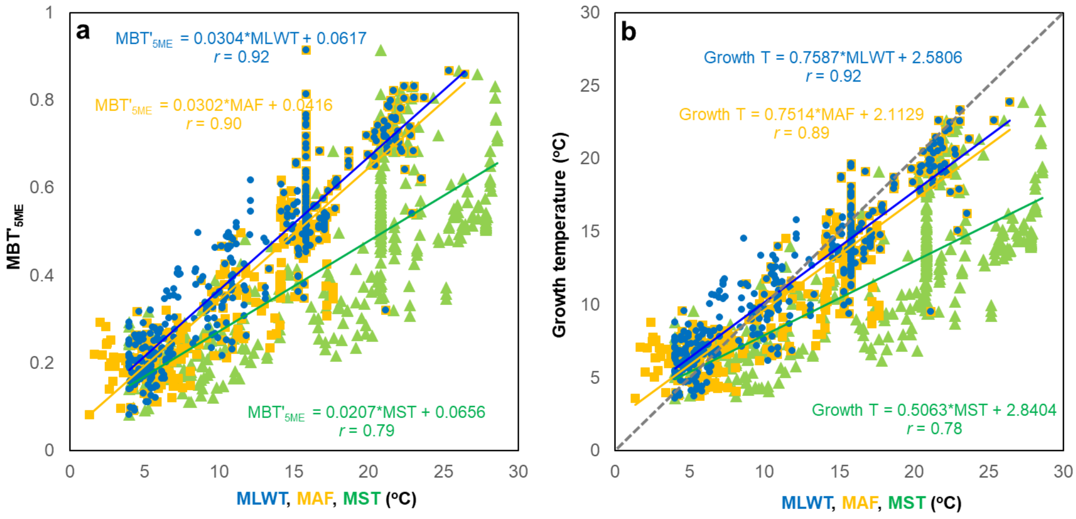

Supplementary Figure 6. **Regressions of MBT'$_{5ME}$ or brGDGT-inferred growth temperature[23] against meteorological temperature in the sediments of global freshwater lakes[21,24,25,27-38].** MAF: mean temperature above freezing; MLWT: mean lake water temperature; MST: mean summer temperature.

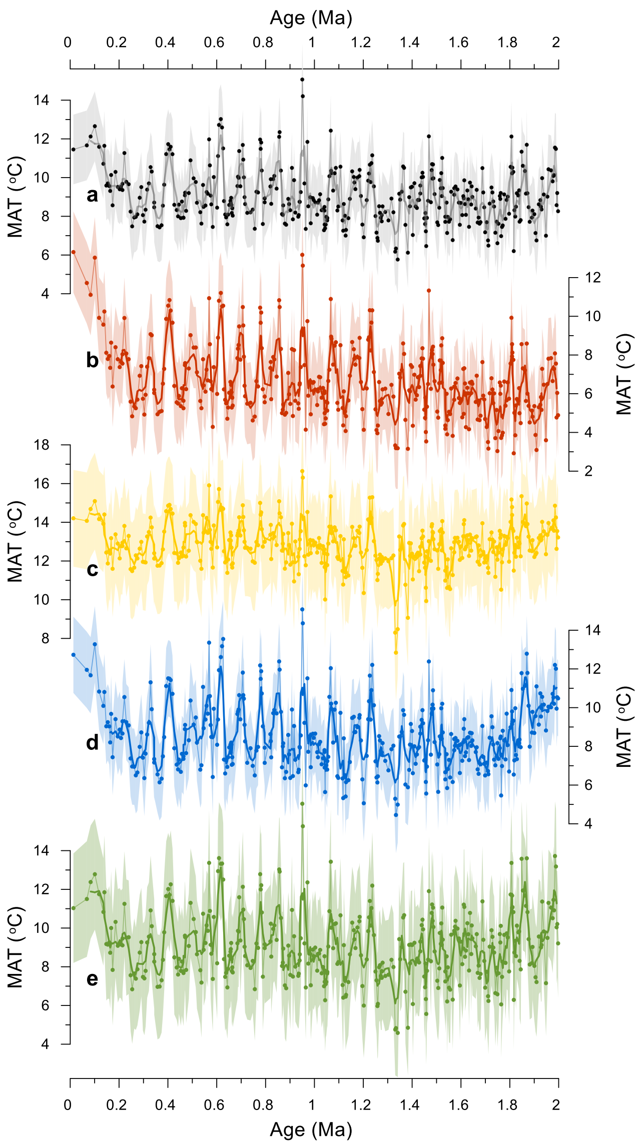

Supplementary Figure 7. **Temperature reconstructions based on various brGDGT calibration equations for the Heqing core. a** The calibration used in this work[23], which can mitigate the mixing effect of 5-methyl and 6-methyl isomer sets. **b** The tropical multivariate linear regression calibration[26]. **c** The Index 1 calibration based on lakes from East Africa and Southwest China[27]. **d** The global Bayesian temperature calibration for lacustrine brGDGTs[24]. **e** The Full set global lacustrine calibration by Raberg et al.[25]. Thick lines indicate 5-point weighted average. Shadings represnent the uncertainties from temperature calibrations.

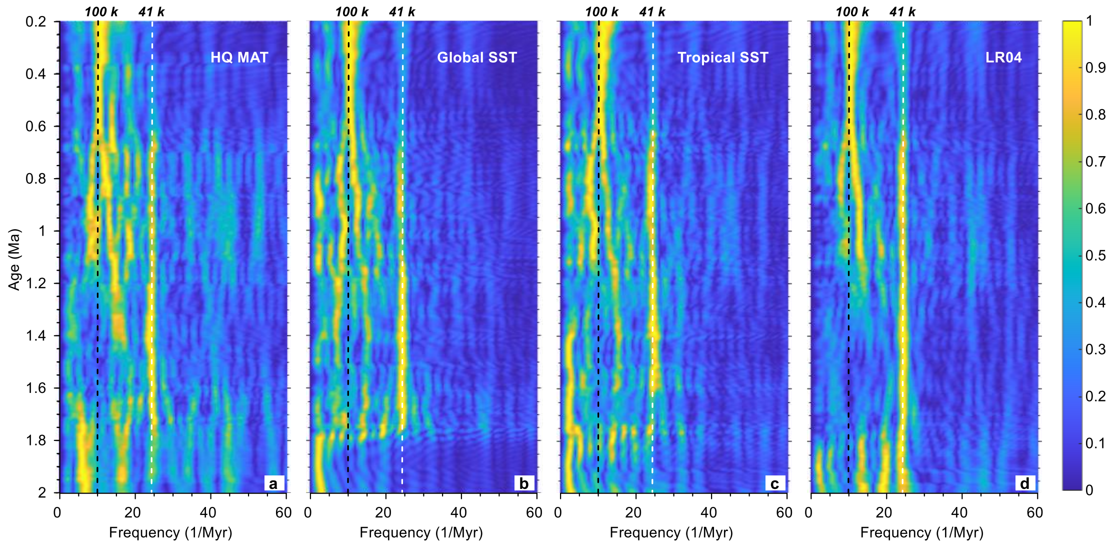

Supplementary Figure 8. **Evolutionary spectrum of Pleistocene temperatures. a** Heqing MAT. **b** The global SST stack[39]. **c** The tropical SST stack[40]. **d** The benthic foraminiferal $\delta^{18}O$[41]. Evolutionary spectrum analysis was performed on *Acycle*[42]. The dominant 100-kyr and 41-kyr periods are indicated by vertical dotted lines.

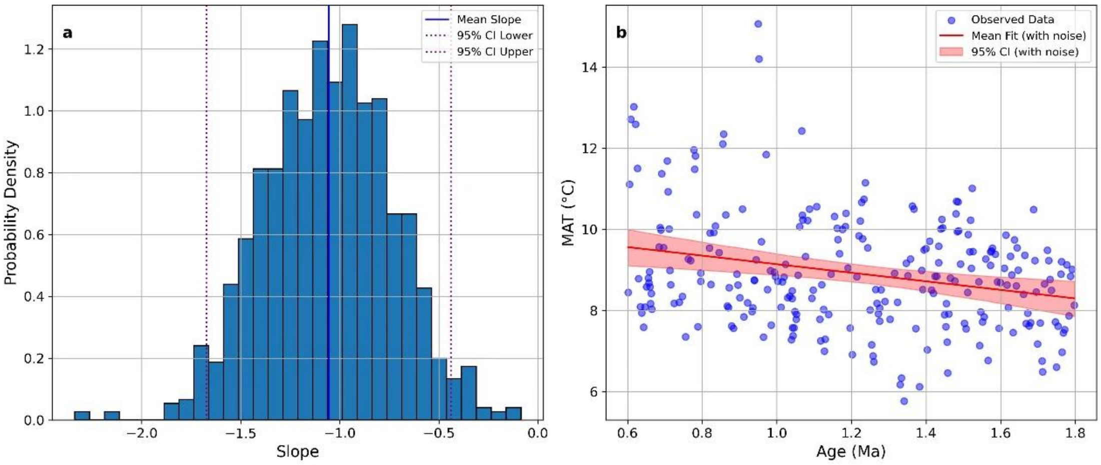

Supplementary Figure 9. **The uncertainty of linear trend for the Heqing temperature record during 1.8–0.6 Ma, based on 1000 times of Monte Carlo simulations with a temperature uncertainty of 1.8 °C. a** Distribution of regression slopes. **b** Regression results with 95% confidence interval (CI). All slopes were negative as they were calculated from younger to older ages.

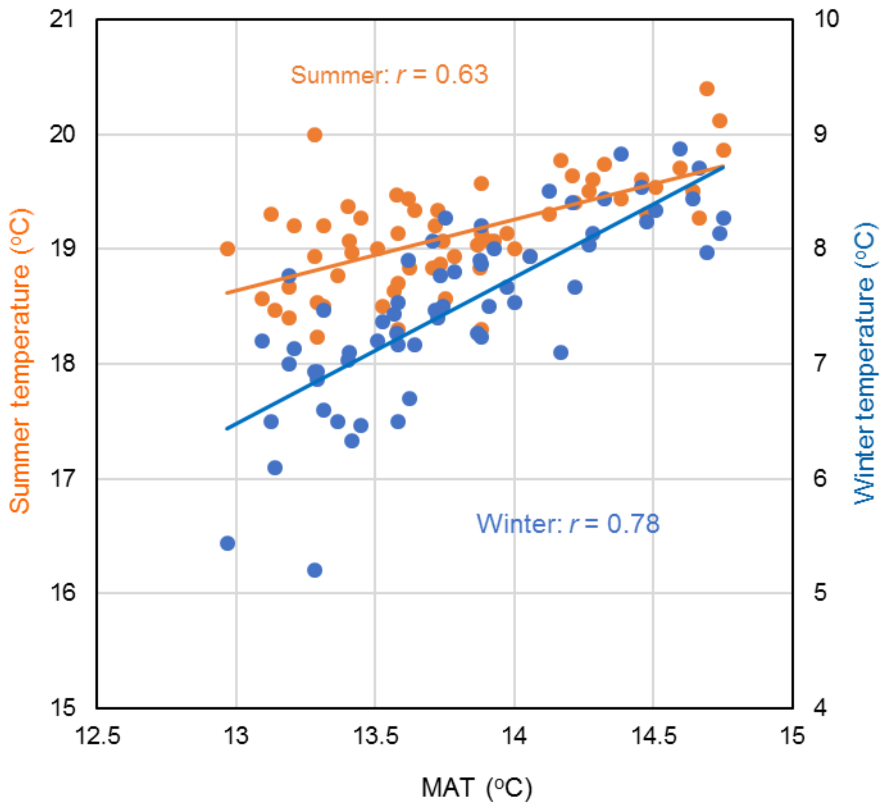

Supplementary Figure 10. **Correlations between seasonal and mean annual temperature (MAT) at Heqing from 1959 to 2022.** Data from the Heqing meteorological station.

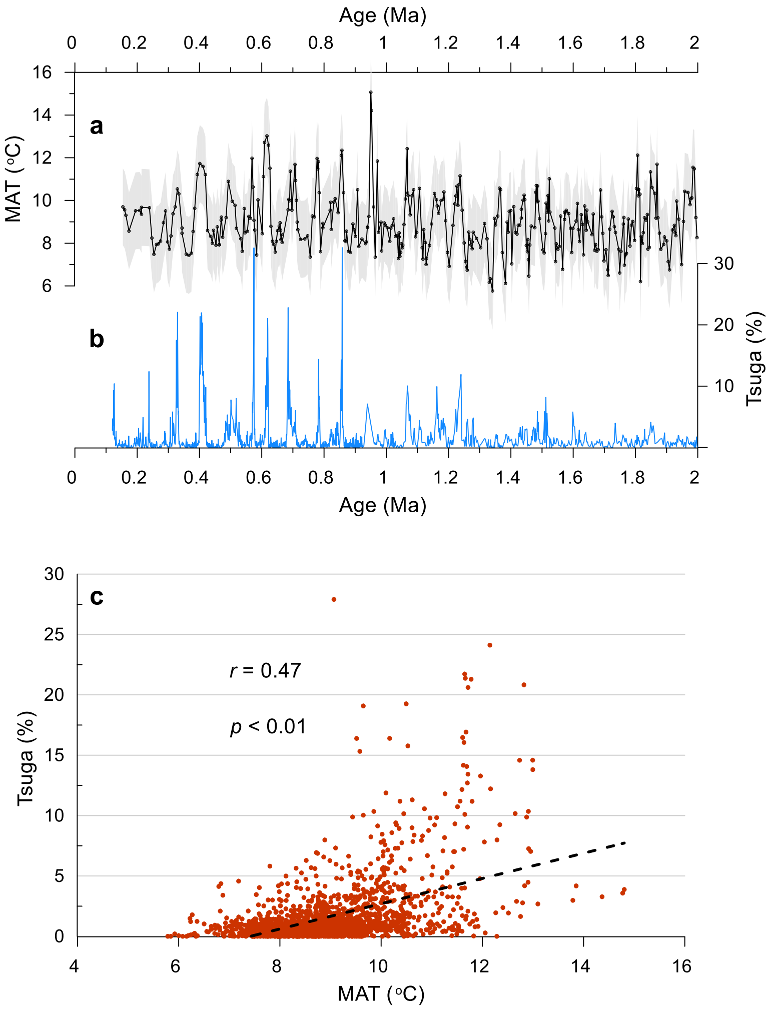

Supplementary Figure 11. **Comparasion of brGDGT-inferred mean annual temperature (MAT) with *Tsuga* pollen content at the Heqing Basin during the past 2 Myr. a** brGDGT-inferred MAT, with the gray shading showing uncertainty from the temperature calibration. **b** *Tsuga* pollen content[43]. **c** Scatter plot of *Tsuga* pollen content against brGDGT-inferred MAT showing a positive correlation between the two proxies.

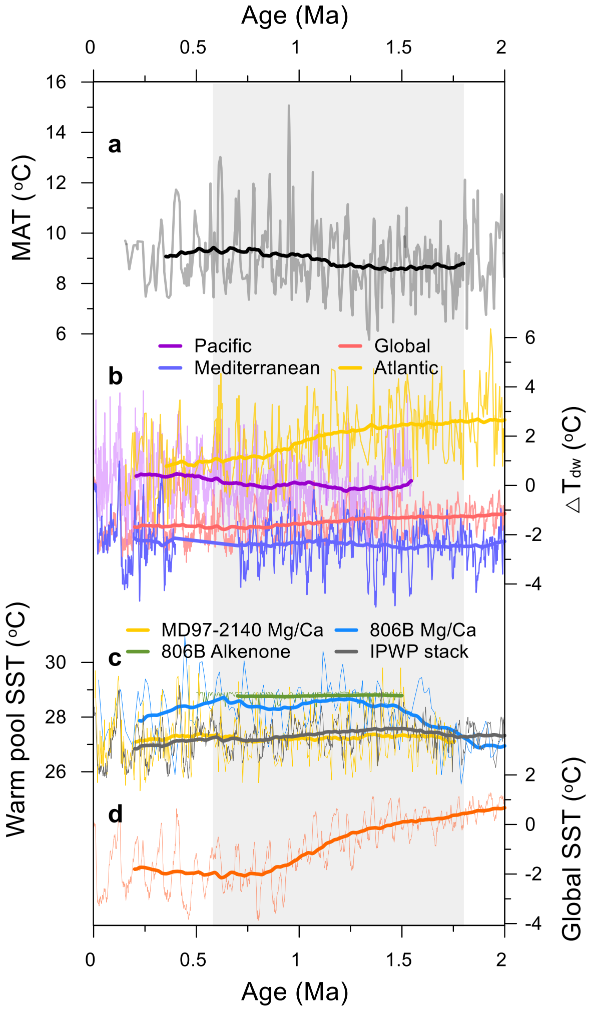

Supplementary Figure 12. **Evolution of Heqing, deep sea, warm pool, and sea surface temperatures during the past 2 Myr. a** MAT inferred from brGDGTs at the Heqing Basin. **b** Deep-water temperature reconstructions from Pacific, Mediterranean sea, Atlantic, and global ocean[44-47]. **c** The Indo-Pacific Warm Pool (IPWP) SST stack[39] and SST records from the core region of IPWP (ODP 806B and MD97-2140)[48-51]. 806B Alkenone: alkenone-based SST reconstruction at 806B; 806B Mg/Ca: SST obtained from Mg/Ca ratios in planktonic foraminifera at 806B; MD97-2140 Mg/Ca: Mg/Ca-based SST reconstruction at MD97-2140. **d** Global SST stack[39]. The thick lines indicate 400-kyr running averages. The gray shading represents the period from 1.8 to 0.6 Ma.

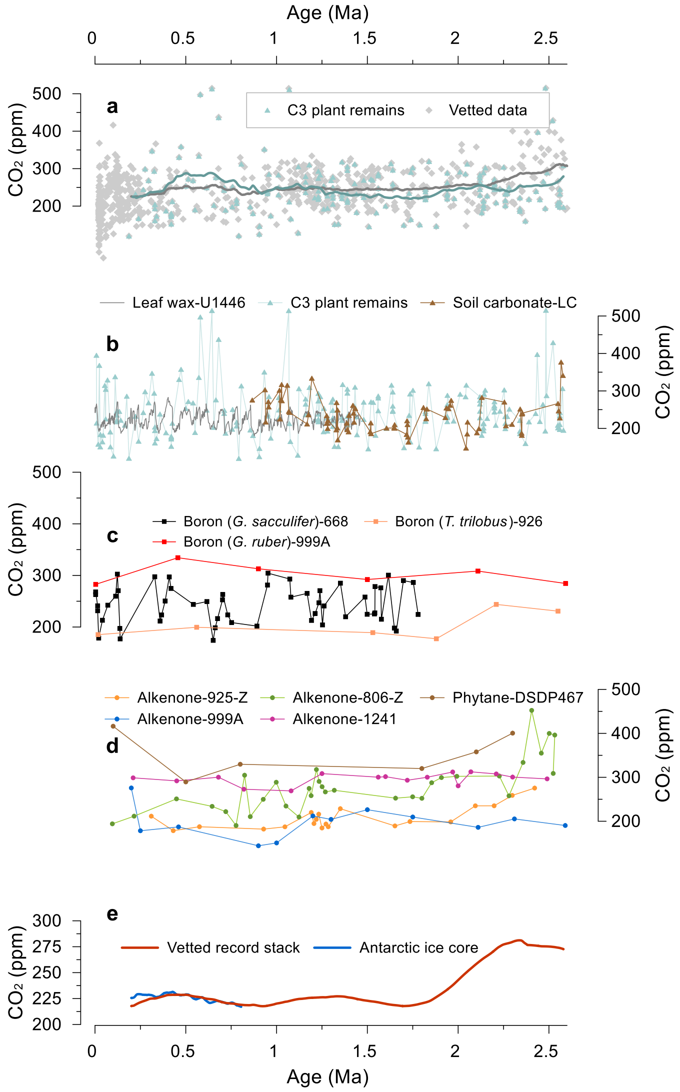

Supplementary Figure 13. **Long CO_2 records and CO_2 stacks during the Pleistocene epoch. a** The compilation of the vetted and modernized data[7] and the CO_2 history inferred from $\delta^{13}C$ of global terrestrial C_3 plant remains[9]. Thick lines indicate 400-kyr smoothing. **b** Vetted long terrestrial CO_2 records inferred from soil carbonate $\delta^{13}C$ in eolian deposits on the Chinese Loess Plateau[8] and the $\delta^{13}C$ of global terrestrial C_3 plant remains[9], and leaf wax $\delta^{13}C$ preserved at IODP U1446[6]. **c** Vetted long CO_2 records reconstructed by boron isotopes ($\delta^{11}B$) in planktic foraminifera *G. ruber*, *G. sacculifer*, and *T. trilobus* at ODP Site 999A, 668, and 926[10-12]. **d** Vetted long CO_2 records calculated from the carbon isotopes of marine phytoplankton biomarkers ($\delta^{13}C_{phytoplankton}$) at ODP Site 806, 925, 999A, and 1241[11,13-15] and DSDP 467[16]. **e** Pleistocene CO_2 stack (400-kyr smoothing) of the 11 CO_2 records in **b-d**. The CO_2 record of Antarctic ice core[17] (400-kyr smoothing) was also plotted.

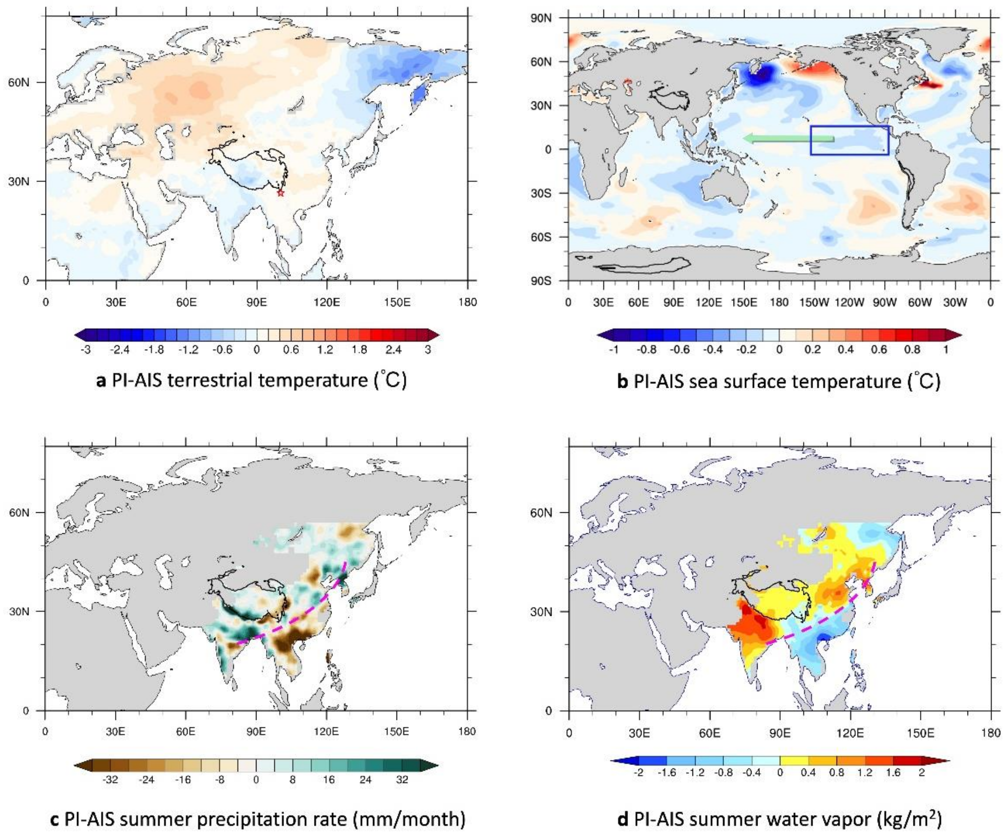

Supplementary Figure 14. **Modelling outputs for the responses of global climate to the expansion of AIS.** **a** Terrestrial temperature difference between PI and AIS experiments showing an overall warming in Eurasia (except Eastern Russia and India) driven by AIS expansion. The red star indicates the Heqing site. **b** Global SST differnece between PI and AIS experiments. The blue box and green arrow highlight a decrease in SST in the eastern tropical Pacific relative to the western tropical Pacific, and thus the strengthening of the zonal SST gradient in the tropical Pacific. **c** Differences in summer precipitation rate over the Asian land monsoon region. **d** Difference in summer water vapor over the Asian land monsoon region. The range of the monsoon region is defined by Wang et al.[52]. The northward penetration of summer precipitation and water vapor in central and northeastern China suggests intensified Asian summer monsoon driven by AIS expansion.

Supplementary References

1. Johnson, T. C. et al. A progressively wetter climate in southern East Africa over the past 1.3 million years. *Nature* **537**, 220–224 (2016).

2. Prokopenko, A. A., Hinnov, L. A., Williams, D. F. & Kuzmin, M. I. Orbital forcing of continental climate during the Pleistocene: a complete astronomically tuned climatic record from Lake Baikal, SE Siberia. *Quat. Sci. Rev* **25**, 3431–3457 (2006).

3. Mackay, A. W. The paleoclimatology of Lake Baikal: A diatom synthesis and prospectus. *Earth Sci. Rev.* **82**, 181–215 (2007).

4. Lindberg, K. R., Daniels, W. C., Castañeda, I. S. & Brigham-Grette, J. Biomarker proxy records of Arctic climate change during the Mid-Pleistocene transition from Lake El'gygytgyn (Far East Russia). *Clim. Past* **18**, 559–577 (2022).

5. Daniels, W. C. et al. Archaeal lipids reveal climate-driven changes in microbial ecology at Lake El'gygytgyn (Far East Russia) during the Plio-Pleistocene. *J. Quat. Sci.* **37**, 900–914 (2022).

6. Yamamoto, M. et al. Increased interglacial atmospheric CO_2 levels followed the mid-Pleistocene Transition. *Nat. Geosci.* **15**, 307–313 (2022).

7. CenCO2PIP Consortium. Toward a Cenozoic history of atmospheric CO_2. *Science* **382**, eadi5177 (2023).

8. Da, J., Zhang, Y. G., Li, G., Meng, X. & Ji, J. Low CO_2 levels of the entire Pleistocene epoch. *Nat. Commun.* **10**, 4342 (2019).

9. Cui, Y., Schubert, B. A. & Jahren, A. H. A 23 m.y. record of low atmospheric CO_2. *Geology* **48**, 888–892 (2020).

10. Hönisch, B., Hemming, N. G., Archer, D., Siddall, M. & Manus, J. F. Atmospheric Carbon Dioxide Concentration Across the Mid-Pleistocene Transition. *Science* **324**, 1551–1554 (2009).

11. Seki, O. et al. Alkenone and boron-based Pliocene pCO_2 records. *Earth Planet. Sci. Lett.* **292**, 201–211 (2010).

12. Sosdian, S. M. et al. Constraining the evolution of Neogene ocean carbonate

chemistry using the boron isotope pH proxy. *Earth Planet. Sci. Lett.* **498**, 362–376 (2018).

13. Zhang, Y. G., Pagani, M., Liu, Z., Bohaty, S. M. & DeConto, R. A 40-million-year history of atmospheric CO_2. *Philos. T. R. Soc. A* **371**, 20130096 (2013).

14. Zhang, Y. G., Pagani, M., Henderiks, J. & Ren, H. A long history of equatorial deep-water upwelling in the Pacific Ocean. *Earth Planet. Sci. Lett.* **467**, 1–9 (2017).

15. Rae, J. W. B. et al. Atmospheric CO_2 over the Past 66 Million Years from Marine Archives. *Annu. Rev. Earth Planet. Sci.* **49**, 609–641 (2021).

16. Witkowski, C. R., Weijers, J. W. H., Blais, B., Schouten, S. & Sinninghe Damsté, J. S. Molecular fossils from phytoplankton reveal secular PCO_2 trend over the Phanerozoic. *Sci. Adv.* **4**, eaat4556 (2018).

17. Bereiter, B. et al. Revision of the EPICA Dome C CO_2 record from 800 to 600 kyr before present. *Geophys. Res. Lett.* **42**, 542–549 (2015).

18. Lenaerts, J. T. M., Vizcaino, M., Fyke, J., van Kampenhout, L. & van den Broeke, M. R. Present-day and future Antarctic ice sheet climate and surface mass balance in the Community Earth System Model. *Clim. Dyn.* **47**, 1367–1381 (2016).

19. Zhu, J. et al. Assessment of Equilibrium Climate Sensitivity of the Community Earth System Model Version 2 Through Simulation of the Last Glacial Maximum. *Geophys. Res. Lett.* **48**, e2020GL091220 (2021).

20. Taylor, K. E., Stouffer, R. J., Meehl, G. A. An Overview of CMIP5 and the Experiment Design. *B. Am. Meteorol. Soc.* **93**, 485–498 (2012).

21. Russell, J. M., Hopmans, E. C., Loomis, S. E., Liang, J. & Sinninghe Damsté, J. S. Distributions of 5- and 6-methyl branched glycerol dialkyl glycerol tetraethers (brGDGTs) in East African lake sediment: Effects of temperature, pH, and new lacustrine paleotemperature calibrations. *Org. Geochem.* **117**, 56–69 (2018).

22. Qiang, X., Xu, X., Zhao, H. & Fu, C. Greigite formed in early Pleistocene lacustrine sediments from the Heqing Basin, southwest China, and its paleoenvironmental implications. *J. Asian Earth Sci.* **156**, 256–264 (2018).

23. Wang, H. et al. New calibration of terrestrial brGDGT paleothermometer

deconvolves distinct temperature responses of two isomer sets. *Earth Planet. Sci. Lett.* **626**, 118497 (2024).

24. Martínez-Sosa, P. et al. A global Bayesian temperature calibration for lacustrine brGDGTs. *Geochim. Cosmochim. Acta* **305**, 87–105 (2021).

25. Raberg, J. H. et al. Revised fractional abundances and warm-season temperatures substantially improve brGDGT calibrations in lake sediments. *Biogeosciences* **18**, 3579–3603 (2021).

26. Zhao, B. et al. Evaluating global temperature calibrations for lacustrine branched GDGTs: Seasonal variability, paleoclimate implications, and future directions. *Quat. Sci. Rev.* **310**, 108124 (2023).

27. Zhao, C. et al. Possible obliquity-forced warmth in southern Asia during the last glacial stage. *Sci. Bull.* **66**, 1136–1145 (2021).

28. Dang, X. et al. Different temperature dependence of the bacterial brGDGT isomers in 35 Chinese lake sediments compared to that in soils. *Org. Geochem.* **119**, 72–79 (2018).

29. Weber, Y. et al. Redox-dependent niche differentiation provides evidence for multiple bacterial sources of glycerol tetraether lipids in lakes. *Proc. Natl. Acad. Sci. U.S.A.* **115**, 10926 (2018).

30. Wang, H. et al. Biomarker-based quantitative constraints on maximal soil-derived brGDGTs in modern lake sediments. *Earth Planet. Sci. Lett.* **602**, 117947 (2023).

31. Qian, S. et al. Rapid response of fossil tetraether lipids in lake sediments to seasonal environmental variables in a shallow lake in central China: Implications for the use of tetraether–based proxies. *Org. Geochem.* **128**, 108–121 (2019).

32. Cao, J., Rao, Z., Shi, F. & Jia, G. Decoupling of water and air temperature in winter causes warm season bias of lacustrine brGDGTs temperature estimates. *Biogeosciences* **17**, 2521–2536 (2020).

33. Yao, Y. et al. Correlation between the ratio of 5-methyl hexamethylated to pentamethylated branched GDGTs (HP5) and water depth reflects redox variations in stratified lakes. *Org. Geochem.* **147**, 104076 (2020).

34. Wang, H., et al. Salinity-controlled isomerization of lacustrine brGDGTs impacts the associated MBT'$_{5ME}$ terrestrial temperature index. *Geochim. Cosmochim. Acta* **305**, 33–48 (2021).

35. Wu, J. et al. Variations in dissolved O2 in a Chinese lake drive changes in microbial communities and impact sedimentary GDGT distributions. *Chem. Geol.* **579**, 120348 (2021).

36. Zhang, C. et al. Quantification of temperature and precipitation changes in northern China during the "5000-year" Chinese History. *Quat. Sci. Rev.* **255**, 106819 (2021).

37. Zhao, B. et al. Development of an in situ branched GDGT calibration in Lake 578, southern Greenland. *Org. Geochem.* **152**, 104168 (2021).

38. Kou, Q. et al. Influence of salinity on glycerol dialkyl glycerol tetraether-based indicators in Tibetan Plateau lakes: Implications for paleotemperature and paleosalinity reconstructions. *Palaeogeogr. Palaeoclimatol. Palaeoecol.* **601**, 111127 (2022).

39. Clark, P. U., Shakun, J. D., Rosenthal, Y., Köhler, P. & Bartlein, P. J. Global and regional temperature change over the past 4.5 million years. *Science* **383**, 884–890 (2024).

40. Herbert, T. D., Peterson, L. C., Lawrence, K. T. & Liu, Z. Tropical Ocean Temperatures Over the Past 3.5 Million Years. *Science* **328**, 1530–1534 (2010).

41. Lisiecki, L. E. & Raymo, M. E. A Pliocene-Pleistocene stack of 57 globally distributed benthic $\delta^{18}O$ records. *Paleoceanography* **20**, PA1003 (2005).

42. Li, M., Hinnov, L. & Kump, L. Acycle: Time-series analysis software for paleoclimate research and education. *Comput. Geosci.* **127**, 12–22 (2019).

43. An, Z. et al. Glacial-interglacial Indian summer monsoon dynamics. *Science* **333**, 719–723 (2011).

44. Elderfield, H. et al. Evolution of ocean temperature and ice volume through the mid-Pleistocene climate transition. *Science* **337**, 704–709 (2012).

45. Rohling, E. J. et al. Sea-level and deep-sea-temperature variability over the past 5.3

million years. *Nature* **508**, 477–482 (2014).

46. Rohling, E. J. et al. Sea level and deep-sea temperature reconstructions suggest quasi-stable states and critical transitions over the past 40 million years. *Sci. Adv.* **7**, eabf5326 (2021).

47. Sosdian, S. & Rosenthal, Y. Deep-sea temperature and ice volume changes across the Pliocene-Pleistocene climate transitions. *Science* **325**, 306–310 (2009).

48. de Garidel-Thoron, T., Rosenthal, Y., Bassinot, F. & Beaufort, L. Stable Sea surface temperatures in the western Pacific warm pool over the past 1.75 million years. *Nature* **433**, 294–298 (2005).

49. McClymont, E. L. & Rosell-Melé, A. Links between the onset of modern Walker circulation and the mid-Pleistocene climate transition. *Geology* **33**, 389–392 (2005).

50. Wara, M. W., Ravelo, A. C. & Delaney, M. L. Permanent El Niño-Like Conditions During the Pliocene Warm Period. *Science* **309**, 758–761 (2005).

51. Medina-Elizalde, M., Lea, D. W. & Fantle, M. S. Implications of seawater Mg/Ca variability for Plio-Pleistocene tropical climate reconstruction. *Earth Planet. Sci. Lett.* **269**, 585–595 (2008).

52. Wang, B., Liu, J., Kim, H. -J., Webster, P. J., & Yim, S.-Y. Recent change of the global monsoon precipitation (1979–2008). *Clim. Dyn.* **39(5)**, 1123–1135 (2011).
